# Separate cortical stages in amodal completion revealed by functional magnetic resonance adaptation

**DOI:** 10.1186/1471-2202-8-70

**Published:** 2007-08-31

**Authors:** Sarah Weigelt, Wolf Singer, Lars Muckli

**Affiliations:** 1 Department of Neurophysiology, Max Planck Institute for Brain Research, Deutschordenstr. 46, D-60528 Frankfurt am Main, Germany; 2 Brain Imaging Center Frankfurt, Schleusenweg 2-16, D-60528 Frankfurt am Main, Germany; 3 Department of Psychology and Center for Cognitive Neuroimaging CCNi, 58 Hillhead Street, University of Glasgow, Glasgow G12 8QB, UK

## Abstract

**Background:**

Objects in our environment are often partly occluded, yet we effortlessly perceive them as whole and complete. This phenomenon is called visual amodal completion. Psychophysical investigations suggest that the process of completion starts from a representation of the (visible) physical features of the stimulus and ends with a completed representation of the stimulus. The goal of our study was to investigate both stages of the completion process by localizing both brain regions involved in processing the physical features of the stimulus as well as brain regions representing the completed stimulus.

**Results:**

Using fMRI adaptation we reveal clearly distinct regions in the visual cortex of humans involved in processing of amodal completion: early visual cortex – presumably V1 -processes the local contour information of the stimulus whereas regions in the inferior temporal cortex represent the completed shape. Furthermore, our data suggest that at the level of inferior temporal cortex information regarding the original local contour information is not preserved but replaced by the representation of the amodally completed percept.

**Conclusion:**

These findings provide neuroimaging evidence for a multiple step theory of amodal completion and further insights into the neuronal correlates of visual perception.

## Background

**Figure 1:**
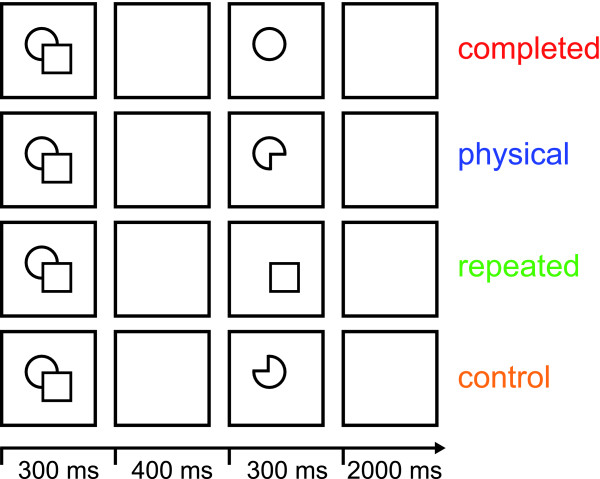
**Experimental procedure**. An experimental trial began with the presentation of one composite stimulus for 300 ms, which was followed by a blank screen for 400 ms. Then the test stimulus was presented for 300 ms, followed by a blank screen for 2,000 ms. The test stimulus was either the completed shape ('completed'), the physical shape of the occluding stimulus ('physical'), a repetition of the occluding shape ('repeated'), or the physical shape rotated in plane around 180 degrees ('control'). All single-shape test stimuli were displayed at the exact positions they obtained in the composite stimulus shown before. Note that the composite stimulus – the stimulus that evokes amodal completion – is the same for all experimental conditions. The four experimental conditions are defined by the four different test stimuli. Thus, differences in fMRI signal are only based on differences between the test stimuli.

In our natural environment we often encounter partly occluded objects. However, what we often perceive in a swift and effortless manner is a whole and complete object (see Figure 1 for an exemplar stimulus). Michotte et al. [1] called this phenomenon visual amodal completion. Systematic psychophysical investigations suggest a sequential process of completion including several stages starting with an initial representation of the (visible) physical stimulus features and ending with a completed representation of the stimulus [2,3]. So far, neuroimaging studies of amodal completion concentrated on the latter stage in the completion process, demonstrating that higher visual areas encode completed objects [4-6]. The goal of our study was to investigate both stages of the completion process by localizing both brain regions involved in processing the physical features of the stimulus as well as brain regions representing the completed stimulus.

To identify brain regions representing different stages of the process of amodal completion we employed a functional magnetic resonance (fMRI) adaptation paradigm [7-9]. In a typical event-related fMRI adaptation paradigm two stimuli are presented sequentially [4,10]. In case the two stimuli are functionally similar thus activating the same or highly overlapping neuronal subpopulations this causes a decrease in the fMRI signal to the second stimulus (adaptation). No such decrease in fMRI signal occurs if the two stimuli are functionally dissimilar activating different subpopulations of neurons (no adaptation). Thus, using fMRI adaptation, the functional characteristics of brain regions can be assessed at the level of neuronal subpopulations, even if those are spatially overlapping and not separable with conventional fMRI. Accordingly, fMRI adaptation refers to the signal attenuation in response to the repetition of a stimulus. Cross-adaptation, in contrast, refers to the signal attenuation that is observed (at some cortical level) because the underlying neuronal process can not effectively distinguish the difference of the stimuli and processes them as effectively similar. Two caveats have to be kept in mind while interpreting fMRI-adaptation results: First, fMRI-adaptation is a relative measurement. Even the baseline condition might have some degree of adaptation if it shares stimulus properties with the experimental conditions. Second, cross-adaptation can also occur along stimulus dimensions that are unpredictable by the experimenter [11]. Therefore, the stimulus set as well as the control conditions have to be defined with utmost caution.

In a recent neuroimaging study Rauschenberger and colleagues [12] made use of an fMRI cross-adaptation paradigm to reveal the neuronal substrate of amodal completion. A stimulus that elicits amodal completion ('pictorial-occlusion prime') was presented either for 100 ms ('brief exposure') or for 250 ms ('long exposure'). The stimulus was masked and then followed by either a stimulus that matched the physical properties ('notched-disk probe') or by a stimulus that matched the completed percept of that stimulus ('complete-disk probe'). Focusing on the temporal unfolding of the completion process, they showed that after brief exposure of the pictorial-occlusion prime the fMRI signal to the notched-disk probe was adapted, while at long exposure of the pictorial-occlusion prime the fMRI signal to the complete-disk probe was adapted. This effect was true for early (V1, V2, V3, V4v) as well as higher visual areas (lateral occipital complex, LOC; ref [13]). The authors conclude that with brief exposures only the representation of the physical stimulus properties is represented in all visual areas. In contrast, at long exposures the physical and the completed interpretation seem to be represented concurrently. Thus, this study is valuable in showing that the amodal completion process takes time to evolve. However, the study is inconclusive with respect to the question of the different roles of early and late cortical processing stages for the process of completion.

In order to address this issue we designed our study in a way that allowed us to directly compare brain activity related to the sensation of the physical properties of the stimulus and brain activity correlated with the perception of the completed shape. First, we used line drawings instead of filled shapes to maximize any possible adaptation effects for early visual cortex. It has been shown that fMRI-adaptation effects in early visual cortex can be elicited by small high-contrast black-and-white elements [14,15]. Second, we presented the second stimulus – whether it was a physical or a completed interpretation – at the very same retinal location it was displayed as part of the stimulus eliciting amodal completion [see Figure 1 and Additional file 1 for the complete stimulus set]. Third, we did not use a masking stimulus because a masking stimulus might lead to additional, potentially conflicting processing of visual information. Fourth, we presented our stimuli for 300 ms to ensure that the stimuli were indeed amodally completed. It has been shown that at a presentation rate of > 200 ms composite stimuli produce priming effects equivalent to their completed interpretation [2].

More specifically, an experimental trial within our rapid-event related design began with the presentation of one composite stimulus for 300 ms, which was followed by a blank screen for 400 ms (see Figure 1). Then the test stimulus was presented for 300 ms, followed by a blank screen for 2000 ms. The test stimulus was either the completed shape ('completed'), the physical shape of the occluding stimulus ('physical'), a repetition of the occluding shape ('repeated'), or the physical shape rotated in plane around 180 degrees ('control'). Note that the composite stimulus – the stimulus that evokes amodal completion – is the same for all experimental conditions. The four experimental conditions are defined solely by the four different test stimuli. Thus, differences in fMRI signal are only based on differences between the test stimuli.

Following the logic of fMRI adaptation and cross adaptation described above we computed the contrast physical < completed in order to localize brain areas that process the physical shape (but not the amodally completed shape) and the contrast completed < physical in order to localize brain areas that process the completed percept (but not the physical shape). In other words, a brain region representing the physical stimulus should display a decreased (cross-adapted) fMRI signal when the physical shape follows the composite stimulus; in contrast, a brain region representing the completed percept should display a decreased (cross-adapted) fMRI signal when the completed shape follows the composite stimulus.

Using such an fMRI-adaptation design we are able to demonstrate a differential effect for early visual cortex, presumably V1 in comparison to higher visual area LOC. We demonstrate that the physical features (local contours) of the stimulus are processed in primary visual cortex. In contrast, higher visual areas such as the LOC no longer represent the original contour constellations but only the segregated and fully completed shapes. We argue in favour of a multiple step model in amodal completion involving different cortical areas.

## Results and discussion

### Brain regions representing the completed stimulus

**Figure 2:**
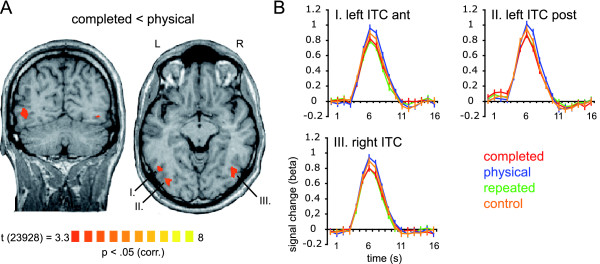
**Brain regions representing the completed stimulus**. *Panel A *displays the contrast map averaged across subjects showing regions responding significantly less to the completed stimulus in comparison to the physical stimulus (contrast completed < physical; p < 0.05, corrected for multiple comparison using a cluster size threshold). The statistical map is superimposed on a coronal and an axial slice of one subject. *Panel B *displays event-related deconvolved BOLD fMRI responses (beta weights, averaged across trials and subjects) from the regions displayed in A reported against time for each of the experimental conditions: completed [red], physical [blue], repeated [green], and control [orange]. Time point 1 = trial onset. Error bars correspond to standard errors of the mean. Abbreviations: ITC, inferior-temporal cortex; post, posterior; ant, anterior.

To identify brain regions encoding the completed stimulus we computed the contrast completed < physical (see 'Methods' for a detailed description of data analyses). The group analysis revealed three regions in inferior temporal cortex (ITC), two in the left hemisphere and one in the right hemisphere (Talairach coordinates [16]: left ITC posterior, x, y, z: -35, -78, -2; left ITC anterior, x, y, z: -47, -63, -4; right ITC, x, y, z: 42, -66, -7). These regions showed significantly (p < 0.05, corrected) less increase of fMRI signal to the completed stimulus in comparison to the physical stimulus (adaptation). Figure 2 displays the contrast map superimposed on two brain slices (Figure 2A) and the event-related BOLD fMRI signal time courses of the corresponding regions (Figure 2B).

We performed additional analyses within these so defined regions-of-interest (ROIs; see Methods). To test for the adaptation to the repetition of the occluding shape ('repeated') we computed the classical adaptation contrast repeated < control (note that this contrast is orthogonal to the ROI-defining contrast). We found that these regions demonstrate a significantly decreased fMRI signal to the repeated stimulus in comparison to the control stimulus (Table 1). This finding reassures us that the observed decreased fMRI signal to the completed stimulus in contrast to the physical stimulus is most likely based on cross-adaptation. Furthermore, all three pre-defined regions displayed a significant adaptation effect for the completed condition in contrast to the control condition (Table 1). However, none of the contrasts physical < repeated, physical < control, completed < repeated showed a significant result (Table 1). We also computed the same contrast that defined the ROI (completed < physical) across voxels within the ROI (across time points). This contrast turned out to be significant at p < 0.000001 (Note that the higher significance level is expected because the selection criteria was identical to this contrast). Furthermore, we calculated single-subject maps for the contrast completed < physical [see Additional file 2]. Since there are no activation patterns for this contrast observable in early visual areas we rule out the possibility that the lack of completion effects in early visual areas is due to individual differences. In sum, three regions in inferior temporal cortex displayed an adapted fMRI signal to the completed and the repeated and no adaptation to the physical and the control condition. We thus conclude that these regions represent the completed and the repeated stimuli.

**Table 1:** ROI-based analysis

	***Regions of interest***							
	Left ITC ant		Left ITC post		right ITC		V1	

**Contrasts**	*P*	*t*	*p*	*t*	*p*	*t*	*p*	*t*

physical < completed	-	-	-	-	-	-	0.00*	3.65
completed < physical	0.00*	5.34	0.00*	6.7	0.00*	5.34	-	-
physical < repeated	0.00^§^	-7.61	0.00^§^	-6.35	0.00^§^	-6.05	0.85	0.19
physical < control	0.01^§^	-2.68	0.01^§^	-2.54	0.03^§^	-2.16	0.08	1.76
completed < repeated	0.02^§^	-2.27	0.73	0.344	0.48	-0.70	0.00^§^	-3.46
completed < control	0.01*	2.66	0.00*	4.15	0.00*	3.18	0.06	-1.88
repeated < control	0.00*	4.93	0.00*	3.81	0.00*	3.89	0.12	1.57

**Figure 3:**
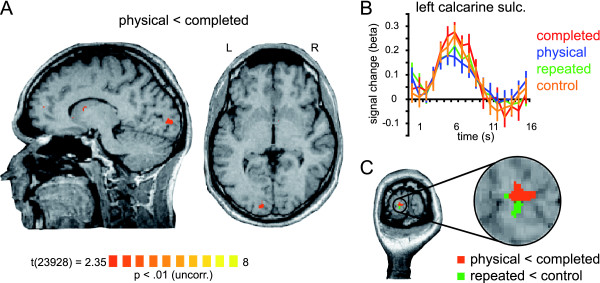
**Brain region representing the physical stimulus**. *Panel A *displays the contrast map averaged across subjects showing a region responding significantly less to the physical stimulus in comparison to the completed stimulus (contrast physical < completed; p < 0.01, uncorrected). The statistical map is superimposed on a sagittal and an axial slice of one subject. *Panel B *displays the event-related deconvolved BOLD fMRI responses (beta weights, averaged across trials and subjects) from this region reported against time for each of the experimental conditions: completed [red], physical [blue], repeated [green], and control [orange]. Time point 1 = trial onset. Error bars correspond to standard errors of the mean. Abbreviations: sulc, sulcus. *Panel C *depicts two contrast maps superimposed on a coronal slice of one subject and a close-up of the activation pattern. The orange contrast map shows the same region as in panel A (contrast physical < completed). The green contrast map shows a region responding significantly less to the repeated stimulus in comparison to the control stimulus (contrast repeated < control).

Statistical values for different pairwise comparisons that were computed on the pre-defined ROIs. Degrees of freedom are 23,928. * p < 0.05 with t > 0; ^§ ^p < 0.05 with t < 0. See Figures 2 and 3 and the results section.

Our results are in congruence with previous neuroimaging [4,5], EEG [6], and MEG [17,18] studies that demonstrated completion effects in higher visual areas. More precisely, these fMRI studies identified a region in inferior temporal cortex, namely the lateral occipital complex, LOC [13], to be involved in visual completion processes [4,5]. The reported Talairach coordinates suggest a perfect match to our results.

But beyond simply confirming that the LOC is involved in the completion process, our data suggest that the LOC represents the fully completed object exclusively. It was not in the scope of the current study to investigate cases when a composite stimulus is present, but no amodally completed shape is perceived (e.g. due to very short presentation times). That is, we can only say that in cases when amodal completion is perceived, the LOC represents the amodally completed object.

While previous investigations used shapes which where occluded by gratings [4,5], we employed shapes which were occluded by shapes. In addition to previous neuroimaging studies our stimulus material allowed us to directly compare the amodally completed shape ('completed') with the actually presented shape ('physical'). Demonstrating fMRI adaptation to the amodally completed shape but not to the actually presented shape, we are able to conclude that at the level of LOC only the amodally completed percept is represented in the visual system. Information regarding the originally presented shape is no longer present. This finding corroborates the assumption by Sekuler and Palmer [2] that the initial representation of the physical properties of the stimulus is not preserved as such at higher processing levels (at least not in the shape processing pathway) but is replaced by the representation of the amodally completed object.

Most notably, the adaptation effect to the completed stimulus relies only on that specific quarter of the stimulus which is actively completed. That is, the completed percept and the originally presented shape share three quarters of their areas and still they are processed fundamentally different in LOC. These data suggest that at the level of the LOC the local contour information available in the picture is no longer present but has been replaced by a representation of the completed percept. In sum, our data suggests that the LOC is the first stage in cortical processing where the fully completed object is represented.

### Brain regions representing the physical stimulus

We performed the contrast physical < completed in order to localize brain regions processing the physical features of the stimulus but not the completed percept. Our group analysis yielded only one region in the calcarine sulcus of the left hemisphere (Talairach coordinates: x, y, and z, -12, -90, -0) that showed a significantly (p < 0.01, uncorrected) decreased fMRI signal to the physical stimulus in comparison to the completed stimulus (adaptation; Figure 3A). Additional ROI-based analyses of this region revealed that the contrast physical < completed is significant at p <0 .0003 (Table 1). Till now it has been a challenge to observe adaptation effects in primary visual cortex and successful paradigms employed rather long and intense adaptation phases [19]. Since we wanted to address early as well as higher visual areas in one experiment, the parameters for our short-term-adaptation design had to be adjusted to suit both cortical stages. Thus, we expected the effects for the early visual areas to be smaller compared to other studies investigating V1. We also expected the adaptation effects in V1 to be smaller compared to the adaptation effects detected in higher visual areas which have been shown to be robust using a variety of paradigms [4,5,7,20]. However, taking into account that our experimental conditions differed only in a quarter of the area of the stimuli presented, the observed adaptation to the physical stimulus is striking. That is, three quarters of the contour and the area of the two stimuli (physical and completed) match completely and the observed adaptation effect can only be due to the fourth quarter being different. In this regard, the demonstrated adaptation effect is quite strong. Furthermore, we computed the contrast map repeated < control and identified a region in the calcarine sulcus of the left hemisphere that displayed the classical adaptation effect. Both regions, i.e. the one showing adaptation to the repetition of the covering stimulus (in comparison to the control stimulus) and the one showing adaptation to the uncompleted physical stimulus (in comparison to the completed) are adjacent to one another and are both in early visual cortex, presumably V1 (Figure 3C).

**Figure 4:**
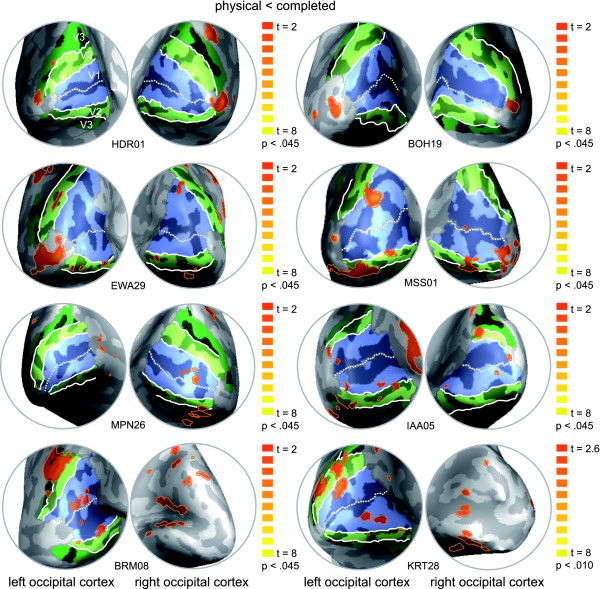
**Single-subject analysis and retinotopic mapping**. Medio-posterior views on the inflated left and right occipital cortex of eight subjects are shown. Retinotopic areas (V1 [light blue], V2 [light green], V3 [dark green]) and individual contrast maps (contrast physical < completed [orange]; p < 0.045; degrees of freedom = 2,666) are superimposed.

We further speculated that high inter-subject variability of anatomical structures might be one cause for the general difficulty to observe adaptation effects in V1 and for our difficulty in particular to observe these effects in the right hemisphere. In order to have a more detailed look onto the observed adaptation effects in early visual cortex we conducted single-subject analyses to compare the individual results to the retinotopy. That is, we computed the contrast physical < completed for each individual subject and superimposed the resulting contrast maps onto the individually defined retinotopic maps (Figure 4). Most notably, in all of our subjects we identified regions in early retinotopic areas of both hemispheres that showed a significantly (p < 0.05) lower fMRI signal for the physical stimulus in contrast to the completed stimulus (adaptation). The exact localization of these regions, however, varied between subjects as expected due to the high inter-subject variability of anatomical structures in the occipital lobe. In four subjects the region was confined to the area representing the fovea, in the other four subjects the region was clearly confined to V1, however at a more eccentric localization. Smaller regions that showed a decrease of fMRI signal for the physical stimulus in contrast to the completed stimulus were also found in V2 (in four subjects bilateral, in two subjects only on one hemisphere). Additional activations were also found in other regions (see Figure 4: e.g. HDR01, right hemisphere and EWA29, left hemisphere, presumably V3A, V3B), however these varied substantially across subjects. Stimuli evoking amodal completion naturally also contain depth information, so we might speculate that also areas processing depth cues are involved in representing these stimuli (e.g. areas of the dorsal pathway -V3a, ref. [21]). Further investigations are needed to clarify this issue.

Thus, the high inter-subject variability explains in part the small effects in our group-analysis. In sum, the single-subject analysis confirms our group-analysis on a more powerful level in detecting adaptation effects in early visual cortex. Here we provide neuroimaging evidence for the processing of the physical features of a stimulus that induces amodal completion. We found regions in early retinotopic areas to demonstrate adaptation of activation to the physical stimulus but not to the completed stimulus (both following a composite stimulus that elicits amodal completion). Importantly, we demonstrate that these regions in early visual cortex also show a clear adaptation effect to the repeated stimulus but no adaptation to the control stimulus (both following a composite stimulus that elicits amodal completion). Since the control stimulus was identical to the physical stimulus but rotated in plane, we conclude that early visual cortex encodes the local contours of the stimulus that induces amodal completion.

Since we presented our composite stimuli in the center of the visual field, the exact localization with respect to early visual areas V1/V2 seems difficult. Nevertheless, our data fits well into a current working model of area V1: One of the major functions of this area is decomposing the visual field into short line segments of different orientations. Line segments carry information about edges and contours. Thus, V1 is said to provide an initial two-dimensional approximation of the shape of a stimulus. We are confident that our data supports the conclusion that the local contours of the stimulus that induces amodal completion are represented in V1.

In the following text passage we will discuss our findings in comparison to the study by Rauschenberger et al. [12]. While this study investigated amodal completion with a focus on temporal dynamics of the completion process using two different presentation times, we used only one presentation time (300 ms). Thus, we are only able to compare our results to the ones obtained by Rauschenberger et al. with the long exposure of 250 ms. In congruence with Rauschenberger et al. [12] we find early as well as higher visual areas to be involved in the processing of stimuli eliciting amodal completion. However, our study allows us to extend this conclusion by demonstrating a differential functional profile for early (V1) in comparison to higher (LOC) visual areas. While Rauschenberger et al. [12] found evidence for a representation of the notched and the completed shape concurrently in all (early and late) visual areas, we were able to reveal a distinctive pattern for early in contrast to late visual areas. In contrast to Rauschenberger we used high-contrast line drawings instead of red/green squares and we presented the test stimuli at the exact retinal location they obtained in the composite stimulus. Both manipulations were chosen to optimize the visual presentation for early visual areas. Furthermore, Rauschenberger et al. [12] used a strong visual backward masking. To our knowledge, it is not known at which levels of processing the mask leads to interferences with the process of amodal completion. Also their probe stimuli consisted of composite stimuli that again triggered the process of amodal completion. Our test stimuli were designed according to the original psychophysical work by Sekuler and Palmer [2], and trigger no additional process of completion. Further differences between the Rauschenberger study [12] and our study concern e.g. magnetic field strength (they 1.5 T, we 3 T) and number of trials per condition (they 48, we 144). In sum, we think that we have carefully selected parameters in order to optimize for statistical power and we have now succeeded to show a differential effect between V1 and LOC on the basis of our different stimulation procedure.

### The process of amodal completion

One reasonable interpretation of our data suggests that amodal completion is a process that involves distinctive different processing in two cortical stages: first, the detection and processing of the local contour information of the stimulus without the completion and second, the representation of the fully completed shape without the representation of the original stimulus. That only the physical features of an occluded object are initially present in the visual system was first suggested by Sekuler and Palmer [2]. They also speculated that this initial representation must be linked to its specific retinal location. Our findings corroborate this assumption. By demonstrating that the physical features of the stimulus are encoded in retinotopic areas of the visual brain we confirm this first prediction. Furthermore, in their comprehensive study, Sekuler and Palmer [2] demonstrated that completed interpretations of composite stimuli can be primed at different positions within the visual field. Thus, they concluded that in a later stage of processing where representations can be more spatially invariant the completed objects are represented (irrespective of their position in the visual field). We show that the fully completed object is represented in the LOC. Moreover, in previous neuroimaging studies, it was found that this area encodes perceived shapes irrespective of their position in the visual field [20,22,23]. It still needs to be directly tested whether also amodally completed objects are represented in the LOC in a position invariant fashion.

If early visual cortex, presumably V1, processes the initial local contour of the stimulus and the fully completed object is encoded in LOC, where does the actual completion process occur? We can only speculate on this issue: After detecting the local contours the basic task for the visual system is to assign the contours to the different objects. In case of occlusion the occluding contour has to be assigned to the occluding object and not to the object being occluded. The missing contour of the object being occluded can then be completed based on Gestalt-rules. Our experiment was not designed to identify this complex computational process. We rather focused on the start and end points of the completion process. Thus, based on our data we are not able to make any inferences about where the computations occur. However, we would like to mention results proposing that this computational effort might take place in area V2. Supporting evidence for this interpretation stems from recent studies using single cell recordings in monkeys [24-26] and work by Zhaoping [27] introducing a corresponding computational model. The electrophysiological investigations demonstrate that the problem of border-ownership is resolved in visual area V2 [24,25]. Area V2 combines stereoscopic cues with Gestalt-rules to represent the borders of 2D figures as if the figures were objects in 3D space [26]. Zhaoping [27] suggests that these computations are based on intracortical interactions in area V2. Furthermore, Bakin and colleagues [24] found that neurons of area V2 signal partially occluded contours by flank facilitation. Furthermore, it has been shown that area V4 is crucial for midlevel form vision and contour integration, and that V4 lesions impair the discrimination of illusory borders [28]. Thus, this area of the ventral visual pathway might also integrate the amodally completed contours. However, there is psychophysical evidence that amodal completion does not only involve contour completion, but also surface and even volume completion [29], which appears to be solved at the level of the LOC.

Our data, taken together with previous findings, are in line with the interpretation of a feed-forward model for the cortical processes underlying amodal completion: First, local contour information is extracted and processed in primary visual cortex. Second, real contours are assigned to different objects in area V2 and missing contours are filled-in on the basis of basic Gestalt-rules (probably V2/V4). Finally, the fully completed object is represented in the LOC. The model suggests a serial order of processing steps. Evidence for such a serial processing comes from behavioural experiments [2,3] as well as recent MEG studies [18,30]. Using different approaches such as priming and a visual search task, both behavioural investigations agree that the representation of the completed shape is preceded by a representation of the local contour information [2,3]. This finding is corroborated by the MEG studies revealing shorter latencies for a primed stimulus that contains only the local contour information of a composite stimulus in contrast to a primed completed shape [18,30]. Another MEG study investigated contextual influences in amodal completion [31]. Also this study found earlier components to be primarily responsive to physical stimulus attributes, while later components resembled the integration of physical stimulus attributes to global objects.

Thus, a feedforward model can explain our results, however, also more complex models involving feedback connections [6] are still under debate that also give credit to the finding that even higher cognitive functions (i.e. visual short term memory) can interfere with the process of amodal completion [32].

In conclusion, our data suggests that the initial representation of the physical properties of the stimulus might still be preserved at the level of V1 but are ultimately replaced at the higher processing level of LOC.

## Conclusion

In the present study we provide neuroimaging evidence for a two stage model of amodal completion. Using fMRI adaptation we are able to demonstrate that the physical features (local contours) of the stimulus eliciting amodal completion are processed in early visual cortex, presumably V1. In contrast, higher visual areas such as the LOC do not represent the original contour constellations but only the segregated and fully completed shapes. Our data are in line with a serial process evolving over time although the temporal evolvement was not the focus of this study. In sum, our study complements psychophysical investigations and electrophysiological findings towards a more complete picture of the cortical processes underlying amodal completion.

## Methods

Ten human subjects (aged 17–32 years, mean 27 years) with normal or corrected-to-normal vision took part in this study. All gave their informed written consent to the procedure in accordance with institutional guidelines and the Helsinki declaration [35]. Each subject participated in the amodal completion study and also underwent standard retinotopic mapping to assess the boundaries of retinotopic cortical areas V1, V2 and V3 (see ref. [33,34] for a detailed description of the procedure).

For the amodal completion study three different geometrical shapes – line-drawings of a circle, a square, and a diamond – formed the basis of the stimulus set. By pairing two of these shapes various composite stimuli were created [see Additional file 1 for the complete stimulus set]. In total, twenty-four different composite stimuli were created. In twelve of the stimuli the occluding shape was a complete shape [line one, Additional file 1] and in the other twelve stimuli the occluding shape was a notched shape [line three, Additional file 1]. This manipulation was introduced to leave room for the interpretation of the occluded stimulus. Importantly, since the physical stimulus was always a notched stimulus and the amodally completed stimulus was always a completed stimulus, we thus made sure that the occluding stimulus (the 'repeated') was in half of the cases similar to the physical stimulus and in the other half similar to the completed stimulus.

Furthermore, thirty-six different stimuli were created depicting line-drawings of single shapes [lines 4–8, Additional file 1]. For each composite stimulus four different single-shape stimuli were created that served as test stimuli (see Figure 1). The stimuli were displayed at the centre of the screen as black line-drawings on a white square subtending 300 × 300 pixels (approximately five degrees of visual angle). The background of the screen was set to gray. Throughout the whole experiment a red fixation cross was present at the centre of the screen and subjects were instructed to maintain fixation. The fixation was small enough (subtending approx. 0.5 deg visual angle) not to overlap with the contours of the stimuli.

Visual stimuli were delivered under computer control using custom-made software (StimulDX, Brain Innovation, Maastricht, The Netherlands) to a high-luminance projector. The image was projected onto a screen that was fixed to the head coil and could be viewed by the subjects through a tilted mirror. Each experimental run began and ended with a fixation period of 10 s. The experimental block in between consisted of 36 experimental trials per condition and 36 fixation trials. For a detailed description of an experimental trial see 'Background' and Figure 1. Subjects performed four experimental runs within one scanning session. Thus, each experimental condition was presented 144 times total. The order of presentation of the experimental trials was counterbalanced across runs per subject. That is, trials from each condition (fixation included) were preceded (2 trials back) equally often by trials from each other condition.

Blood oxygenation level-dependent fMRI [36] was performed with a standard birdcage head coil on a 3 T Siemens Trio Scanner (Siemens, Erlangen, Germany) at the Brain Imaging Center, Frankfurt, Germany. A gradient-recalled echo-planar imaging sequence was used with the following parameters: 16 axial slices; repetition time (TR) = 1,000 ms; echo time (TE) = 40 ms; flip angle (FA) = 60°; field of view (FOV) = 210; slice thickness = 5 mm; in-plane resolution = 3 × 3 mm^2^; gap thickness = 0.5 mm. Slices were oriented to achieve a total coverage of the occipital and temporal lobes. Each scanning session included the acquisition of a high-resolution magnetization-prepared rapid-acquisition gradient echo sequence (TR = 2,250 ms; TE = 2.62; FA = 9°; FOV = 256; slice thickness = 1 mm; In-plane resolution = 1 × 1 mm^2^) for coregistration and anatomical localization of functional data.

Data analysis and visualization was performed using the BrainVoyager QX software package (Brain Innovation, Maastricht, The Netherlands). The first four volumes of each event-related run were discarded to preclude T1 saturation effects. Pre-processing of the functional data included motion correction, linear trend removal and temporal highpass filtering at 0.01 Hz, and slice-scan-time correction. Functional 3D data was spatially filtered employing a Gaussian filter with 8-mm kernel for the group analysis only. One subject had to be excluded from further analyses due to strong head movements. For each subject the functional and structural 3-D data sets were transformed into Talairach coordinate space [16]. The recorded high-resolution anatomical scans were used for surface reconstruction.

Our rapid event-related fMRI study used closely spaced trials, leading to a substantial overlap in the resulting hemodynamic responses. Nevertheless, when the pre-cautions of a balanced randomization are taken (two-back randomization, see above), the underlying hemodynamic responses can be assessed by deconvolution [37]. A deconvolution analysis estimates the hemodynamic response function for each trial on the basis of a general linear model (GLM). Twenty predictors (one predictor per volume) were defined to cover the temporal extent of a typical hemodynamic response. Statistical analyses were conducted volume-based and included a multi-subject fixed-effects GLM as well as single-subject fixed-effects GLMs. All GLMs were computed on the peak points of the fMRI-signal. GLMs were corrected for serial correlation and normalized to percent signal change. That is, the time course of a voxel or a region-of-interest is normalized in such a way that the mean signal value is transformed to a value of 100 with the individual values fluctuating around the mean as percent signal deviations. Thus, the reported signal change beta weights directly provide an estimate of the actual percent signal change.

For the statistical map completed < physical we performed a cluster-size thresholding to correct for multiple comparisons [38,39]. We employed a plugin for Brain Voyager QX by Fabrizio Esposito (Brain Innovation, Maastricht, The Netherlands) that makes use of a Monte-Carlo simulation (1000 iterations). The original map was thresholded at p = 0.000968, t(23,928) = 3.3. The estimated cluster size threshold was 322 voxels (1 × 1 × 1 mm^3^). Consequently, the multisubject statistical map in Figure 2 is thresholded at p < 0.05, corrected for multiple comparisons.

For the statistical map physical < completed no correction for serial correlation was applied. Since to-date it has been a challenge to observe adaptation effects in primary visual cortex and successful paradigms employed rather long and intense adaptation phases [19]. Since we wanted to address early as well as higher visual areas, parameters for our short-term-adaptation design had to be adjusted to suit both cortical stages. Thus, we expected the effects for the early visual areas to be small.

Following the logic of fMRI adaptation described in the 'Background' we computed the contrast physical < completed in order to localize brain areas that process the physical shape (but not the amodally completed shape) and the contrast completed < physical in order to localize brain areas that process the completed percept (but not the physical shape). In other words, a brain region representing the physical stimulus should display a decreased (adapted) fMRI signal when the physical shape follows the composite stimulus; in contrast, a brain region representing the completed percept should display a decreased (adapted) fMRI signal when the completed shape follows the composite stimulus.

To ensure that the identified adaptation profile is truly based on 'adaptation' and not simply a lower fMRI signal we looked at the response profiles of the pre-defined regions in more detail. First, we computed the contrast repeated < control. This contrast is also called the basic adaptation contrast since it tests whether the identified region displays a conventional adaptation effect to a repeated stimulus. Second, we additionally computed the contrasts physical < repeated, physical < control, completed < repeated, and completed < control. Third, we also computed the same contrast that defined the ROI also within the ROI – however, since this computation is not statistically independent, it is only of descriptive use. We report t-statistics, degrees of freedom and p-values (Table 1).

## Competing interests

The author(s) declare that they have no competing interests.

## Authors' contributions

SW conceived and designed the study, carried out the data collection, analyzed the data and drafted the manuscript. LM designed the study, contributed to data analysis, and co-drafted the manuscript. WS contributed to study design and co-drafted the final version of the manuscript. All authors read and accepted the final version of the manuscript.

## Supplementary Material

Additional file 1 The complete stimulus set. The complete stimulus set comprising all twenty-four composite and thirty-six test stimuli are presented. Click here for file

Additional file 2 Individual maps for completed < physical. Medio-posterior views on the inflated left and right occipital cortex of eight subjects are shown. Retinotopic areas (V1 [light blue], V2 [light green], V3 [dark green]) and individual contrast maps (contrast completed < physical [orange]; p < 0.045; degrees of freedom = 2,666) are superimposed. Click here for file
